# Epigenetic regulation of NKG2D ligand and the rise of NK cell-based immunotherapy for cancer treatment

**DOI:** 10.3389/fonc.2024.1456631

**Published:** 2024-08-05

**Authors:** Raj Kumar, Romi Gupta

**Affiliations:** ^1^ Department of Biochemistry and Molecular Genetics, The University of Alabama at Birmingham, Birmingham, AL, United States; ^2^ O’Neal Comprehensive Cancer Center, The University of Alabama at Birmingham, Birmingham, AL, United States

**Keywords:** epigenetics, NK cell, acetylation, methylation, clinical trial

## Abstract

Epigenetic modifications influence gene expression and effects cancer initiation and progression. Therefore, they serve as diagnostic and prognostic biomarkers and potential therapeutic targets. Natural Killer (NK) cells, integral to the innate immune system, exhibit anti-tumor effect by recognizing and eliminating cancerous cells through the balance of activating and inhibitory ligands. Understanding the epigenetic regulation of NK cell ligands offers insights into enhancing NK cell-mediated tumor eradication. This review explores the epigenetic modifications governing the expression of activating NKG2D ligands and discusses clinical trials investigating NK cell-based immunotherapies, highlighting their potential as effective cancer treatment strategies. Case studies examining the safety and effectiveness of NK cell therapies in different cancer types, such as acute myeloid leukemia (AML) and non-small cell lung cancer (NSCLC), demonstrate promising outcomes with minimal toxicity. These findings underscore the therapeutic prospects of epigenetic modulation of NKG2D ligands and NK cell-based immunotherapies as effective cancer treatment strategies. Future research in the advancement of personalized medicine approaches and novel combination therapies with NK cell will further improve treatment outcomes and provide new therapeutic options for treating patients with various types of cancer.

## Introduction

Epigenetic changes regulating gene expression are those that are independent of alterations in the DNA sequence. These changes can be regulated by numerous factors (1). Epigenetic regulators mediating epigenetic changes have been shown to play a pivotal role in cancer initiation and progression by regulating the expression of tumor suppressor genes (TSGs) and oncogenes (2). Moreover, these epigenetic changes impact DNA repair mechanisms, potentially leading to genomic instability and driving tumorigenesis (3, 4). They also contribute to maintaining the stemness of cancer cells (5) and regulate responses to therapy (6). Therefore, they are used as biomarkers for cancer diagnosis and prognosis and represent promising targets for cancer therapy. Understanding the role of epigenetic regulators and epigenetic modifications in cancer development is essential for unraveling the complexity of the disease and developing effective targeted therapeutic strategies.

Natural Killer (NK) cells is a kind of white blood cell and represents crucial components of the immune system. NK cells are a part of the innate immune system and play an important role in regulating the immune response to viral infections, as well as in recognizing and destroying infected or abnormal cells, such as cancer cells (7). NK cells possess the ability to identify and target cancer cells without prior sensitization (8). They express receptors that recognize specific ligands on cancer cells and, upon recognition, release cytotoxic granules that induce apoptosis, thereby eradicating cancer cells (9).

Cancer cells express both activating and inhibitory ligands, and the balance of these ligands interacting with receptors on the surface of NK cells determines the fate of cancer cells (10). Activating ligands include Natural Killer Group 2D (NKG2D) ligands e.g., Major histocompatibility complex (MHC) class I chain-related protein A (MICA), MHC class I chain-related protein B (MICB), UL16 binding proteins (ULBPs), trigger NK cell activation and promote cytotoxicity upon their expression (11). Another group of activating ligands include DNAX accessory molecule- 1 (DNAM-1) ligands e.g., Nectin-2 (CD112), PVR or cluster of differentiation155 (CD155). DNAM-1 is expressed on NK cells and serve as an activating receptor. It specifically interacts with DNAM-1 ligands expressed on cancer cells and promotes NK cell-mediated cytotoxicity by producing cytokines (12). Inhibitory ligands, for example MHC class I molecules upon interacting with inhibitory receptors on NK cells leads to transmitting signals that prevent NK cell activation and killing of self-cells (13). Thus, the balance between the expression of activating and inhibitory ligands determines NK cell responsiveness to cancer cells. Additionally, NK cells play a role to modulate the overall immune response. It interacts with other immune cells such as macrophages, dendritic cells, and T cells and contributes to the overall regulation of the immune system’s function (14).

Given the important role that NK cells play, potential therapeutic applications of NK cells in cancer treatment are being explored (7). Several clinical trials are ongoing to develop novel therapies based on NK cells for effective cancer treatment. Additionally, previous studies have provided understanding into the epigenetic mechanisms that play a critical role in regulating the expression of NK cell ligands (15–17), affecting NK cell-mediated tumor cell eradication. This article discusses the role of epigenetic modifications that regulate the expression of activating NKG2D ligands and reviews various clinical trials with NK cells, highlighting the effectiveness of NK cell-based immunotherapy as a successful and promising therapeutic strategy for cancer treatment.

## Epigenetic modifications regulating the expression of activating NKG2D

Epigenetic modifications are crucial for regulating gene expression, including the expression of NKG2D ligands (15–17). This section provides a review of various epigenetic modifications that regulate NKG2D ligand expression. The concept of epigenetic regulation of NKG2D ligands and its role in NK-cell mediated antitumor response to eliminate tumor cells was conceived long ago. In a study published in 2011, authors showed that the epigenetic drugs hydralazine, a DNA methylation inhibitor and valproic acid, an inhibitor that belong to class I histone deacetylases (HDACs) and regulator of DNA methylation and demethylation of histones (18) increased the expression of MICA and MICB NKG2D ligands on the target cells. It was via increasing the H3K4 methylation mark at the promoter region of MICA and MICB genes. The increase in expression of MICA and MICB led to an increase in NK cell-mediated cytotoxicity, leading to tumor growth suppression (19).

Since then, several studies have reported that epigenetic modification play an imminent role in regulating the expression of NKG2D ligand. Histone acetylation is a type of posttranslational modification regulated by histone acetyl transferases (HATs) that functions as acetyltransferases and aid in transferring the acetyl moiety of acetyl-CoA to lysine (K) residues on histone. In contrast, HDACs function as deacetylases and remove the acetyl moiety (20). Epigenetic modification involving acetylation is reversible (20). Previous studies as described below have demonstrated that histone acetylation regulates NKG2D ligand expression such as MICA, MICB, and ULBPs, which are critical for the activation of natural killer (NK) cells and immune surveillance against cancer cells. In a study published by Cho et al., authors found that treatment of lung cancer cells with a selective HDAC1/2 inhibitor FK228, also known as Romidepsin, led to the induction NKG2D ligands expression both at mRNA and protein level (21). This resulted in increased cytotoxicity of NCI-H23 cells to NK cells (21). Similar results were obtained when the expression of both HDAC1 or HDAC2 was inhibited, suggesting that suppressing HDAC1 and HDAC2 expression both genetically and pharmacologically leads to activation of NKG2D ligands and facilitate NK cell-mediated anti-tumor effects in lung cancer (21).

In another study published by Mormino et.al., authors demonstrated that histone deacetylase HDAC8 regulate the expression of NKG2D ligands. They further showed that pharmacological inhibition of HDAC8 using the small molecule inhibitor PCI-34051 led to the upregulation of ULBP1, H60A, and RAE1 NKG2D ligands in glioma cells by significantly increasing H3K4me3 levels, resulting in an increase in NK-mediated cytotoxicity (22). Similar observations were made in other cancer types where authors reported that treatment of Panc89 (a pancreatic carcinoma cell line) and PC-3 (a prostate carcinoma cell line) with Valproic acid (VPA), a histone deacetylase inhibitor, significantly enhanced the expression and/or release of the several NKG2D ligands such as ULBP-2, MICA, MICB (23).

Additionally, interesting results were obtained in non-small cell lung cancer (NSCLC) cell line NCI-H23 cells, where ionizing radiation and HDAC inhibitors (Apicidin, SAHA, or TSA) in combination synergistically increased both the expressions of NKG2D ligands and sensitivity of NSCLC cell line to NK cells-NK-92, enhancing tumor eardication (24). Moreover, in colon carcinoma and sarcoma cells, treatment with HDAC inhibitor Entinostat, a class I HDAC1 and HDAC3 inhibitor, led to increase in expression of NKG2D ligand MICA and MICB, enhancing cytotoxicity of NK cells against tumor cells (25). Thus, several studies have confirmed that histone acetylation/deacetylation plays a crucial role in regulating the expression of NKG2D ligand and NK cell mediated tumor clearance in multiple cancer types.

Apart from histone acetylation, histone methylation has also been shown to regulate the expression of NKG2D ligands. In a previous study by Bugide et al., it was demonstrated that Enhancer of zeste homolog 2 (EZH2), which functions as histone-lysine N-methyltransferase, serves as a transcriptional repressor of NKG2D ligands. Pharmacological or genetic inhibition of EZH2 led to an increase in the expression of NKG2D ligands and enhanced eradication of hepatocellular carcinoma (HCC) cell (16). Furthermore, Bugide et al. in another study showed that histone deacetylase 10 (HDAC10) promotes EZH2 recruitment to the chemokine (C-X-C motif) ligand 10 (CXCL10) promoter, resulting in its transcriptional repression. They further demonstrated that CXCL10 is required and sufficient to stimulate NK cell migration and this phenomenon is conserved in EZH2-dependent cancers besides HCC (17 **).**

In a different study performed in acute myeloid leukemia (AML), screening was performed with 70 small molecular drugs in NB4 cell line, an acute promyelocytic leukemia cell line. This screening with small molecular inhibitor showed that lysine-specific demethylase 1 (LSD1) inhibitor tranylcypromine (2-PCPA) hydrochloride significantly up-regulated ULBP2/5/6 expression in the NB4 cells and increased sensitivity of NB4 cells to NK-dependent killing. LSD1 functions to demethylate mono- or di-methyl-lysine 4 or 9 of histone H3 (H3K4me1/me2 and H3K9me1/me2, respectively) by interacting with different proteins (26). This study also demonstrated significant reduction in tumor growth upon combination with human NK cells and LSD1 inhibition (27). These results highlight that methylation regulates NKG2D ligands expression, contributing to immune evasion in acute myeloid leukemia (AML).

Bromodomain and extra-terminal (BET) proteins serve as epigenetic readers of acetylated histones and are also been known to regulate the expression of NKG2D ligands. For example, previous studies have shown that inhibition of BET proteins by JQ1 and I-BET151 or selective BRD4-degrader proteolysis targeting chimera (PROTAC) (ARV-825) leads to an increase in the expression of NKG2D ligand MICA in multiple myeloma (MM) cell lines [RPMI-8226, U266, ARP-1, JJN3, SKO-007(J3)] isolated from MM patients and CD138^+^ MM cells. Increased expression of NKG2D ligand MICA resulted in increased NK cell-mediated cytotoxicity (28). These studies collectively confirm that various epigenetic mechanisms regulate the expression of multiple NKG2D ligand in different cancer cell types, and modulating these mechanisms offers the possibility of enhancing NK-cell based therapy for cancer treatment.

## Clinical trials with NK-cells for cancer therapy

Based on the potential of NK cells to eradicate cancer cells, several clinical trials are testing their effectiveness in cancer therapy. In this phase I clinical trial, the safety and efficacy of NK cells in combination with IgG1 antibodies were examined for the treatment of advanced colorectal or gastric cancers. Adoptive NK cell therapy was utilized alongside trastuzumab- or cetuximab-based chemotherapy for the treatment. After 3 days of administration of IgG1 antibody, patients were administered NK cells three times at triweekly intervals. It was observed that there were no severe adverse effects and the combination therapy was well tolerated. Among the six tested patients, four displayed stable disease (SD) while two showed progressive disease. Notably, three out of four patients with stable disease showed a positive response to the combination therapy, with an overall decrease in tumor size. These outcomes indicate the effectiveness of this combination therapy in treating advanced colorectal or gastric patients, who have been prior treated (29).

Another clinical trial was performed in non-small-cell lung cancer (NSCLC) patients with positive epidermal growth factor receptor (EGFR) mutation. The goal of this trial was to manage acquired resistance (advanced NSCLC) to gefitinib and also extend progression free survival (PFS) in the patient. Here, the effectiveness and safety of allogenic CD8 + CD56+ Natural Killer T (NKT) cell immunotherapy in combination with gefitinib for patients with advanced NSCLC with EGFR mutations was explored (30). The results of this trial showed that the treatment used in this study could effectively delay gefitinib resistance also and enhance anti-tumor immune response leading to better clinical outcome (31). Thus, the success of this trial provides the hope for testing NKT cell immunotherapy in context of other EGFR-tyrosine kinase inhibitors (TKIs) and various other types of tumors.

In a separate phase I/IIa clinical trial in patients with stage IV non-small-cell lung cancer, the safety and efficacy of natural killer (NK) cell therapy (SNK01), which were ex vivo activated and expanded were evaluated in combination with pembrolizumab. In this trial, 18 patients with advanced non-small cell lung cancer, who previously failed frontline platinum-based therapy were randomized to receive either pembrolizumab as monotherapy or pembrolizumab in combination with SNK01. The study observed that the objective response rate (ORR) and the 1-year survival rate was higher in patients who received NK combination therapy with pembrolizumab than those who received pembrolizumab as monotherapy (ORR, 41.7% vs. 0%; 1-year survival rate, 66.7% vs. 50.0%). Additionally, the median progression-free survival (PFS) was higher in the SNK01 and pembrolizumab combination group (6.2 months vs. 1.6 months, p=0.001). These results confirm that NK cell combination therapy can be safely used for the treatment of stage IV NSCLC patients who did not respond to platinum-based therapy with least toxicity (32). Based on the success of this trial, another follow-up 2-year study was conducted to determine the long-term effectiveness of the combination therapy. In this study, 20 patients with advanced NSCLC who did not respond to prior frontline platinum-based therapy were included. Patients either received pembrolizumab alone in combination with low-dose (2 × 10^9^cells/dose) or high-dose of SNK01 (4 × 10^9^cells/dose), or just pembrolizumab alone as monotherapy. The results obtained were promising and showed that the 2-year survival rate was higher in the combination group compared to when pembrolizumab was used as monotherapy (58.3% versus 16.7%). They also found that although the median progression-free survival was significantly higher in patients who received pembrolizumab in combination with SNK01 than the ones who receives only pembrolizumab, overall survival, and progression-free survival did not differ between the patients who received low doses or high doses of NK cells (33).

In another trial (NCT03958097), the safety of PD-1 antibody in combination with autologous NK cells was tested for the treating non-small-cell lung cancer patients of stage IIIB/IIIC or IV who did not respond to the first-line platinum-based chemotherapy. In this research, 20 patients received both PD-1 antibody (sintilimab) and NK cells, with a median follow-up time of 22.6 months. The results showed that autologous NK in combination with sintilimab effectively promoted antitumor activity and displayed least toxicity. Thus, this trial suggested that NK cells in combination with sintilimab can be used for treating advanced, mutation-negative NSCLC patients who did not respond to the first-line treatment (34).

In another promising phase I clinical trial, fludarabine/cytarabine followed by 6 infusions of NK cells expanded from haploidentical donors was used to treat 12 patients (median age 60 years) with refractory AML (median 5 lines of prior therapy, median bone marrow blast count of 47%). In this trial, neither graft-versus-host disease (GVHD), nor any toxicity was observed in patients. Results from this study further showed that 7 out of 12 patients (58.3%) achieved response and complete remission (CR) with/without count recovery with 48 days as median time for the best response. The other 5 responding patients got haploidentical transplant from the same donor and were monitored for 52 months. The one-year overall survival (OS) for all 12 patients was observed to be 41.7%, with better outcome noted for patients who achieved a complete response with incomplete count recovery (57.14%), and for those patients who responded and underwent transplantation (60%). In conclusion, in this trial, significant response was observed in refractory AML patients with ex vivo expanded NK-cell administration without adverse effects (35).

Thus, the success of these clinical trials demonstrates that NK-cell based immunotherapies have the potential to serve as effective cancer treatment strategies either alone or in combination with other anti-cancer agents with minimal toxicity.

## Future direction of NK cell therapy

Future directions in NK cell therapy research hold promise for advancing cancer treatment through personalized medicine approaches and novel combination therapies. Similar to CAR-T cells, chimeric antigen receptor (CAR) NK cells engineered to express receptors targeting specific antigens on cancer cells will be a significant progress in this direction. Further future research focusing on optimizing CAR constructs for enhanced specificity, persistence, and efficacy against various cancers can also provide significant benefit in area of cancer therapy. Moreover, tailoring NK cells through genetic editing techniques like CRISPR/Cas9 will offer new opportunities to enhance their targeting capabilities and improve immune recognition of cancer cells. Finally, developing methods to expand and activate NK cells from patients’ own immune cells could enhance efficacy and reduce immune rejection in clinical settings.

Studies have also suggested that combination therapy, whereby combining NK cells with immune checkpoint inhibitors like pembrolizumab or nivolumab, could enhance anti-tumor immune responses. Additionally, conducting trials to explore the synergistic effects of NK cell therapy with cytokines (e.g., IL-2, IL-15) or other immune stimulants to boost NK cell activity and sustain anti-tumor responses will provide beneficial outcomes. Exploring epigenetic modulators to augment NK cell cytotoxicity by enhancing NKG2D ligand expression or reducing immune evasion mechanisms in tumors will be yet another way to enhance the efficacy of NK cell-based therapy. Thus, future trials are certainly needed to explore optimal dosing of the combination agents to achieve higher efficacy with minimum toxicity in patients.

In conclusion, future research directions aimed at advancing personalized medicine approaches and novel combination therapies will help in improving treatment outcomes and providing new therapeutic options for treating patients with various types of cancer.

## Conclusion

In conclusion, epigenetic changes, by regulating gene expression, play an important role in cancer initiation, progression, and determining therapeutic responses. Moreover, epigenetic modifications play key role in regulating NKG2D ligands expression, influencing NK cell-mediated tumor cell eradication. By modulating epigenetic mechanisms, novel therapeutic strategies can be developed to enhance NK cell activity and improve cancer treatment outcomes. Current clinical trials exploring NK cell-based immunotherapy have shown encouraging results, demonstrating both safety and efficacy across various cancer types. Therefore, future treatments employing the combination of epigenetic modulators with NK cell-based immunotherapy will hold great promise and may discover new avenues for personalized and targeted therapeutic approaches. Continued research and clinical trials to advance personalized medicine approaches and novel combination therapies with NK cell holds will undoubtedly lead to further advancements in cancer therapy and improve patient outcomes.

## Author contributions

RK: Writing – review & editing. RG: Writing – original draft, Writing – review & editing, Funding acquisition, Project administration.
